# Highly stretchable polymer-dispersed liquid crystal-based smart windows with transparent and stretchable hybrid electrodes

**DOI:** 10.1039/c8ra07033d

**Published:** 2018-10-30

**Authors:** Jin-Yeong Park, Han-Ki Kim

**Affiliations:** School of Advanced Materials Science & Engineering, Sungkyunkwan University 2066 Seobu-ro, Jangan-gu Suwon Gyeonggi-do 440-746 Republic of Korea hankikim@skku.edu +82-31-290-7410 +82-31-290-7391

## Abstract

We report on highly stretchable polymer dispersed liquid crystal (PDLC)-based smart windows using Ag nanowires (NWs) and conductive PEDOT:PSS hybrid electrodes. By bar coating a Ag NW and PEDOT:PSS mixed ink on a transparent and stretchable polyurethane (PU) substrate, we fabricated highly transparent and stretchable hybrid electrodes with a sheet resistance of 40 ohm per square, an optical transmittance of 82%, and a stretchability of 30% to replace conventional brittle ITO electrode. Bending and stretching tests demonstrated that the mechanical properties of the Ag NW and PEDOT:PSS hybrid electrode were better than those of the ITO/PU sample. The Ag NW/PEDOT:PSS hybrid film was employed as a transparent and stretchable electrode (TSE) in PDLC-based stretchable smart windows, an application that is impossible for brittle ITO-based smart windows. The stretchable PDLC-based smart windows exhibited an on-state transmittance of 56% at an applied voltage of 80 V and an off-state transmittance of 2% at 0 voltage. Unlike an ITO-based PDLC smart window, which is easily broken by stretching, the Ag NW/PEDOT:PSS hybrid electrode-based PDLC smart window was stretched up to 30%. Successful operation of the stretchable PDLC-based smart window indicates that Ag NW/PEDOT:PSS hybrid films are promising TSEs for cost-effective, large area, and stretchable smart windows.

## Abstract 2

Stretchable PDLC window fabricated on the stretchable Ag nanowire and PEDOT:PSS hybrid electrodes.

## Graphical abstract

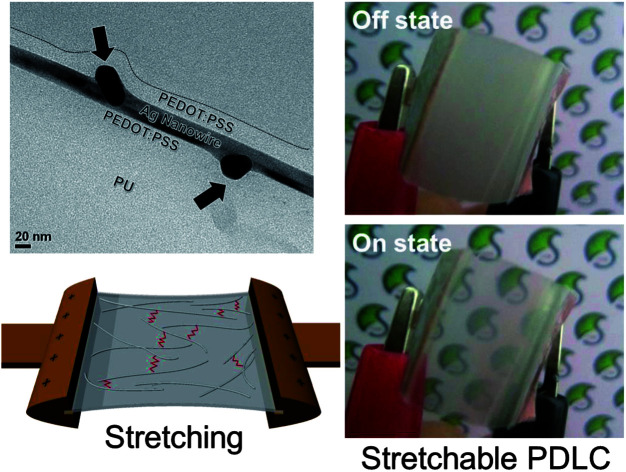

## 1. Introduction

Polymer-dispersed liquid crystal (PDLC)-based smart windows have been widely used due to simple control of transparency by an applied electric field, a simple electrode-sandwiched structure and an easy fabrication process.^1–4^ In typical PDLC-based smart windows, the PDLC layer was sandwiched by a pair of indium tin oxide (ITO)-coated glasses, which were prepared by typical direct current (DC) magnetron sputtering. Because electric fields were applied to liquid crystal (LC) droplets in the polymer through the ITO electrodes, the voltage drop, switching time, and transparency of the PDLC-based smart windows are significantly affected by the electrical and optical properties of ITO films.^5^ In addition, a typical smart window has a large surface area, and the cost of the vacuum-based ITO coating process led to an increase in fabrication cost of large area PDLC-based smart windows. Furthermore, application of sputtered ITO electrodes in flexible or stretchable PDLC-based smart windows is difficult as the brittle ITO film readily cracks when the substrate is extremely flexed or stretched.^6–8^ To replace brittle and high cost ITO electrodes, various transparent and flexible electrodes (TFEs) such as Ag nanowires (NWs), graphene, carbon nanotubes, and conducting polymers have been extensively investigated in flexible PDLC-based smart windows.^9–14^ Although several flexible TFEs showed potential as flexible electrodes for flexible PDLC-based smart window, there have been no reports on stretchable PDLC-based smart windows with stretchable electrodes due to limitations in transparent and stretchable electrode (TSE) materials. The operation of stretchable PDLC-based smart windows requires that the transparent electrodes maintain their transparency and resistivity when the PDLC-based smart windows are stretched by an external force. Although Ag NW networks are promising TFEs for flexible displays and flexible photovoltaics,^15–17^ they cannot be applied in a stretchable PDLC-based smart window as a stretchable electrode due to poor adhesion of Ag NWs with the substrate and disconnections in the short length Ag NW network when the substrate is stretched.^18–21^ Although we report the feasibility of brush-paintable Ag NW : PEDOT:PSS hybrid films as a TSE,^22^ brush painting process had a critical limit to coat uniform TSEs on large area substrate. Therefore, the development of a cost-effective and uniform coating process for TSEs are imperative to realize high performance stretchable and low cost PDLC-based smart windows.

In this work, we investigated the possibility of producing stretchable PDLC-based smart windows using cost effective, transparent, stretchable, and uniformly coated Ag NW and PEDOT:PSS hybrid electrodes coated on 150 μm thick stretchable polyurethane (PU) substrates. Using Ag NW and PEDOT:PSS mixed hybrid ink and a simple bar-coating process, we developed high quality, cost-effective, and uniformly coated TSEs with a low sheet resistance and high transmittance. In addition, we investigated the mechanical flexibility and stretchability of the Ag NW and PEDOT:PSS hybrid electrode in detail and suggested a possible mechanism to explain the high stretchability of Ag NW and PEDOT:PSS hybrid electrodes. Furthermore, we compared the performance of a stretchable PDLC-based smart window fabricated on 100 nm thick sputtered ITO (reference) and 60 nm thick printed Ag NW/PEDOT:PSS hybrid electrodes to show the feasibility of the Ag NW/PEDOT:PSS hybrid electrodes to replace typical ITO electrodes for next generation stretchable smart windows.

## 2. Experimental

### 2.1 Bar coating of Ag NW/PEDOT:PSS hybrid film on a PU substrate

**Fig. 1:**
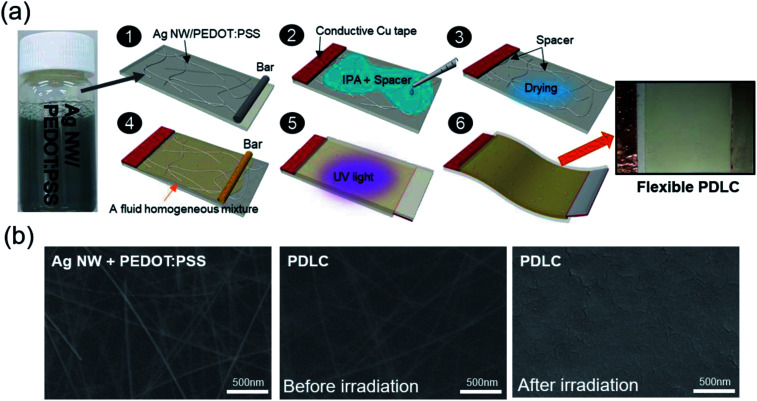
(a) Schematic diagram of PDLC-based smart windows fabrication procedure on stretchable Ag NW/PEDOT:PSS hybrid electrodes. (b) Surface FESEM images of as-printed Ag NW/PEDOT:PSS hybrid electrode (left side) and fluid homogeneous mixture in PDLC solution coated on Ag NW/PEDOT:PSS hybrid electrode before (center) and after (right side) UV irradiation.

We formulated the Ag NW (length 25 ± 5 μm, width 30 ± 5 nm, DI water 0.1%) and conductive PEDOT:PSS (Clevios HTL Solar) mixed ink at a ratio of 20 : 1 as shown in Fig. 1 to print Ag NW/PEDOT:PSS hybrid films on a PU substrate under atmospheric conditions. Pure Ag NWs coated on transparent and stretchable PU substrate showed poor adhesion, poor connectivity, and easy agglomeration during printing process. Therefore, they showed a large deviation in sheet resistance, which is an important parameter for high quality PDLC-based smart windows. Specifically, severe agglomeration of pure Ag NWs in the ink on a hydrophobic PU substrate led to difficulty in forming a uniform Ag NW network. In case of pure PEDOT:PSS films, they showed fairly high sheet resistance in spite of their high optical transmittance and mechanical flexibility. Therefore, we mixed conductive PEDOT:PSS in the Ag NW ink to improve the uniformity of sheet resistance and adhesion of the Ag NW on a PU substrate. Hybrid Ag NW and PEDOT:PSS materials take advantage of both the metallic conductivity of the Ag NW network and the outstanding stretchability of the conducting polymer with high strain failure. In addition, the conducting PEDOT:PSS matrix connected the uncontacted Ag NWs and improved the uniformity of the hybrid electrode *via* the “bridge effect” described in our previous work.^22^ The Ag NW and PEDOT:PSS mixed ink was stirred at 120 rpm for more than 5 hours for uniform mixing of Ag NW and PEDOT:PSS. Then, the Ag NW and PEDOT:PSS mixed ink was coated on the PU substrate using a commercial bar coating system (KP-30000VH) at a constant substrate holder temperature of 70 °C. Mixed ink was uniformly coated on the PU substrate with a size of 300 × 210 mm^2^ using an automatically moving linear bar with a speed of 20 mm s^−1^. After the bar coating process under atmospheric conditions, the residual solvent of the Ag NW and PEDOT:PSS hybrid electrode was removed by heating of samples at a temperature of 70 °C for 30 min.

The electrical and optical properties of hybrid TSEs were measured using Hall measurements (HL5500PC, Accent Optical Technology) and a UV/visible spectrometer (UV 540, Unicam). The flexibility of the bar-coated Ag NW/PEDOT:PSS hybrid electrodes was investigated by measuring resistance change during inner/outer bending. At a fixed bending radius, cyclic inner and outer bending was performed for 10 000 cycles at a frequency of 1 Hz. The resistance changes during sample bending were measured *in situ* through the clamps that hold the edges of the samples. In addition, the stretchability of Ag NW/PEDOT:PSS hybrid TSEs and sputtered ITO electrodes on PU substrates were investigated using a lab-made stretching test system. The surface morphology changes and crack formation of the hybrid TSE and ITO electrode were observed by field emission scanning electron microscopy (FESEM) before and after stretching tests.

### 2.2 Fabrication and evaluation of stretchable PDLC-based smart windows

To show the feasibility of the Ag NW/PEDOT:PSS hybrid film as a promising TSE, we fabricated stretchable PDLC-based smart windows with Ag NW/PEDOT:PSS hybrid electrodes and sputtered ITO films with a size of 20 × 25 mm^2^ as illustrated in Fig. 1(a). Conductive Cu tape was attached on the edge of hybrid TSEs to apply power to the PDLC-based smart window with Ag NW/PEDOT:PSS hybrid TSEs. Then, microsphere spacers (40 μm; GS-240) dispersed in isopropyl alcohol were dropped on the surface of Ag NW/PEDOT:PSS hybrid electrode and dried in air. The gap between two transparent electrodes was maintained by microsphere spacers. Then, the PDLC solution was also coated using bar coater on the Ag NW/PEDOT:PSS hybrid electrode with microsphere spacers. The PDLC solution was made of NOA-65 monomer and E7 in a weight ratio of 1 : 1. After coating of PDLC solution, the other side of the transparent PU substrate coated with the same TSE was placed to form a shifted sandwich, and then both hybrid TSEs were bonded by irradiating with ultraviolet (UV) light having a center wavelength of 365 nm using polymerization induced phase separation (PIPS) method.^23^ The fluid homogeneous mixture in PDLC solution was originally transparent, but it turned into an opaque layer when cured with 365 nm UV light under conditions of 0.5 mW cm^−2^ and 30 minutes.

## 3. Results and discussion

Fig. 1(b) (left side) shows a surface FESEM image of an as-deposited Ag NW/PEDOT:PSS hybrid electrode. Clearly, the Ag NWs formed randomly connected networks in the conductive PEDOT:PSS matrix. The Ag NW/PEDOT:PSS hybrid electrode showed a fairly uniform surface morphology and uniformly distributed Ag NWs due to the well-mixed Ag NW/PEDOT:PSS hybrid ink and uniform bar-coating process.

The center and right-side images in Fig. 1(b) show the surface FESEM images of the bar-coated PDLC solution before and after UV irradiation. A network morphology of Ag NW below the PDLC layer was not observed after 365 nm UV irradiation due to the polymerization of the fluid homogeneous mixture after UV irradiation. The surface morphology of the PDLC layer on the Ag NW/PEDOT:PSS hybrid electrode was very flat and smooth after 365 nm UV irradiation. This indicates that the morphology of the Ag NW/PEDOT:PSS hybrid electrode did not affect the surface morphology of the PDLC layer, but did influence the transparency loss of the PDLC-based smart window.

**Fig. 2:**
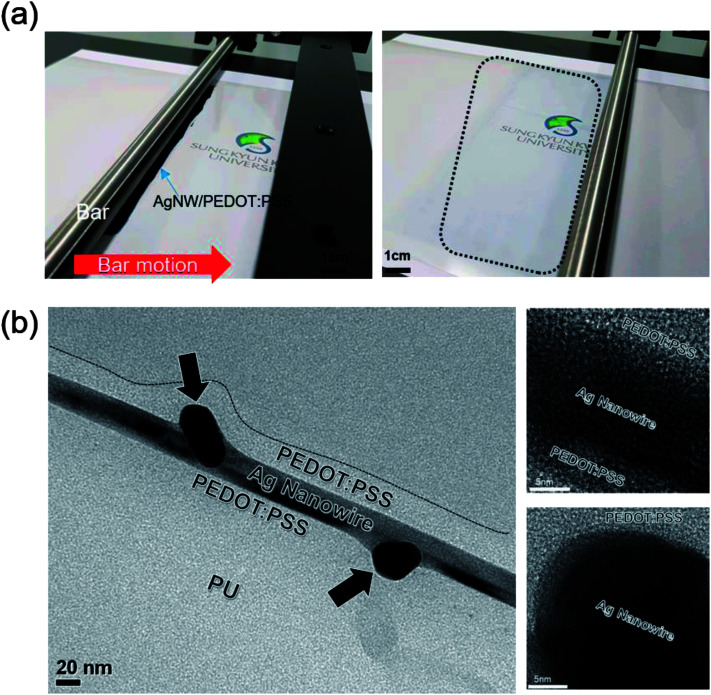
(a) Picture of linear bar coating process with Ag NW/PEDOT:PSS mixed ink on PU substrate. The dark gray solution below linear bar indicating the mixed ink. The dashed area is uniformly coated Ag NW/PEDOT:PSS electrode region. (b) Cross-sectional TEM image of interface between Ag NW/PEDOT:PSS hybrid TSE and PU substrate. Right side images are enlarged Ag NW covered by amorphous PEDOT:PSS conducting layer.

Fig. 2(a) shows the bar-coating process with Ag NW/PEDOT:PSS mixed ink on the PU substrate. Dark gray color solution below a linear bar indicates the Ag NW/PEDOT:PSS mixed ink. After automatic motion of a linear bar, the Ag NW/PEDOT:PSS ink was uniformly coated on the PU substrate (dashed area) due to the uniform shear stress of the linear bar. Unlike simple brush painting,^22^ the automatic bar coating process led to large area coating of Ag NW/PEDOT:PSS ink with a good uniformity. Fig. 2(b) shows a cross-sectional TEM image of the bar-coated Ag NW/PEPOT:PSS hybrid TSE on a PU substrate. It was noteworthy that the Ag NWs were well-mixed or embedded in the conductive PEDOT:PSS matrix without protruding or vertically aligned Ag NWs. Ag NWs were well aligned parallel to the PU substrate due to the shear force of a linear bar moving parallel to the PU substrate. Dark contrast region indicated by arrows is the junction region of the bar-coated Ag NWs. In addition, the enlarge TEM images show that the crystalline Ag NW is well covered by amorphous PEDOT:PSS conducting layer, which act as bridge in current conduction. The uniform and smooth surface morphology is very important for TSEs used in PDLC-based smart windows because light scattering on a rough interface could affect the performance of the PDLC-based smart window.

**Fig. 3:**
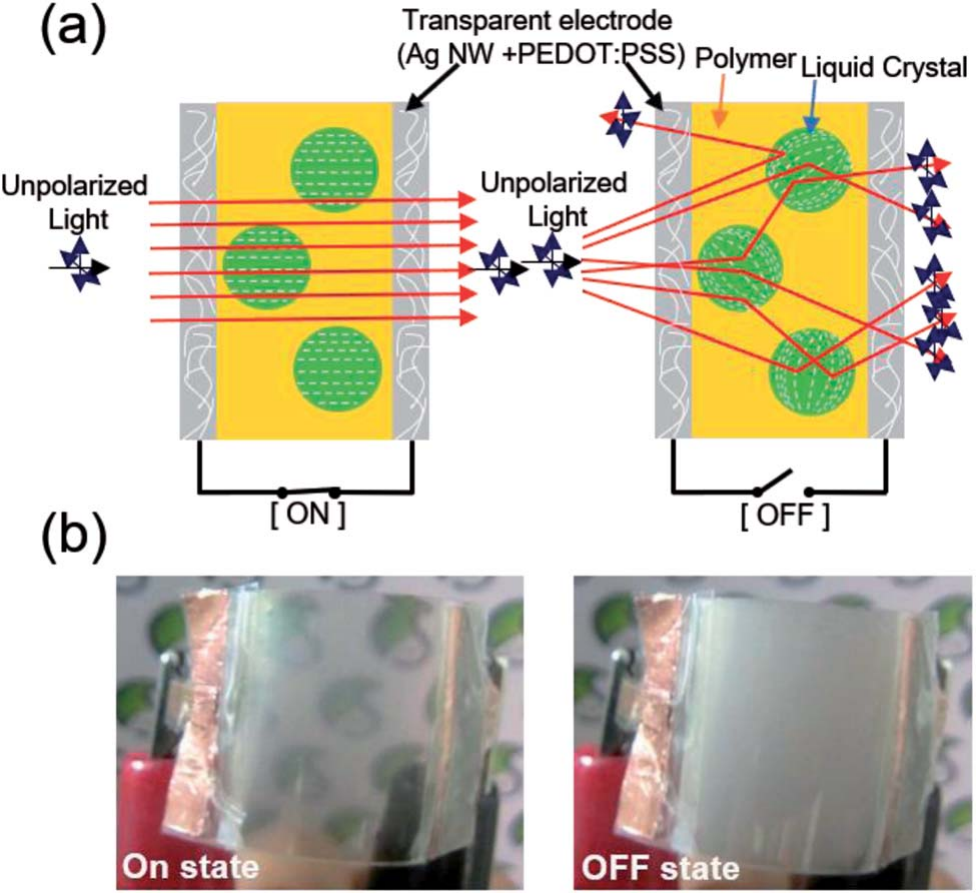
(a) Schematic of the operation mechanism of PDLC-based stretchable smart window with Ag NW/PEDOT:PSS hybrid electrodes. (b) The pictures show the on and off state of PDLC-based smart window with Ag NW/PEDOT:PSS hybrid TSEs.

Fig. 3 exhibits a schematic of the structure and operation principle of Ag NW/PEDOT:PSS hybrid TSEs-based PDLC smart window. Like the typical ITO-based PDLC smart window, the stretchable PDLC smart window with Ag NW/PEDOT:PSS hybrid TSEs covered both sides of the PDLC layer.^24^ As shown in Fig. 3(a), the LC molecules were arranged parallel to the applied electric field direction when a voltage was applied to the Ag NW/PEDOT:PSS hybrid TSEs. When the refractive indices of the LC molecules and the polymer are the same, the incident light passes through the cell, and the PDLC layer becomes transparent. A LC director inside each LC droplet has a random orientation when the external voltage is removed. In this state, the incident light is scattered due to the refractive index difference between the LC molecules and the polymer, which results in an opaque and slightly ivory color layer as shown in right side of Fig. 3(b).^25–27^

The bar-coated Ag NW/PEDOT:PSS hybrid TSEs showed a low sheet resistance from 20 to 200 ohm per square depending on bar-coating speeds. In bar-coating process, speed of the linear bar motion could influence on the electrical of the Ag NW/PEDOT:PSS hybrid electrodes because the amount of mixed-ink used in the bar-coating process depends on the speed of the linear bar motion. In this work, we used Ag NW/PEDOT:PSS hybrid TSEs with a sheet resistance of 40 ohm per square considering the optical transmittance of the hybrid TSE.

**Fig. 4:**
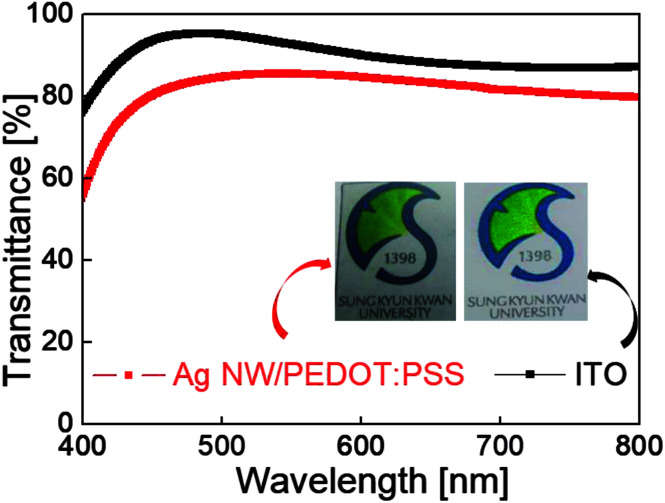
Optical transmittance of Ag NW/PEDOT:PSS hybrid TSE and ITO film coated on PU substrate in the visible wavelength region (400–800 nm).

Fig. 4 compares the optical transmittance of the Ag NW/PEDOT:PSS hybrid TSE and typical ITO electrode coated on a PU substrate in the visible wavelength region between 400 and 800 nm. Although the Ag NW/PEDOT:PSS hybrid TSE showed a lower optical transmittance than that of the ITO electrode, an optical transmittance of 82% is enough to fabricate stretchable PDLC-based smart windows for buildings or automobiles. Table 1 compares the electrical and optical properties of the bar-coated Ag NW/PEDOT:PSS hybrid TSE and sputtered ITO films on a stretchable PU substrate.

**Table 1:** Comparison of transmittance and sheet resistance of the bar-coated Ag NW/PEDOT:PSS electrode and sputtered ITO electrode

	Ag NW/PEDOT:PSS electrode	ITO electrode
Transmittance at 550 nm [%]	81.58	89.74
Sheet resistance [ohm per sq.]	40 ± 5	40 ± 3

**Fig. 5:**
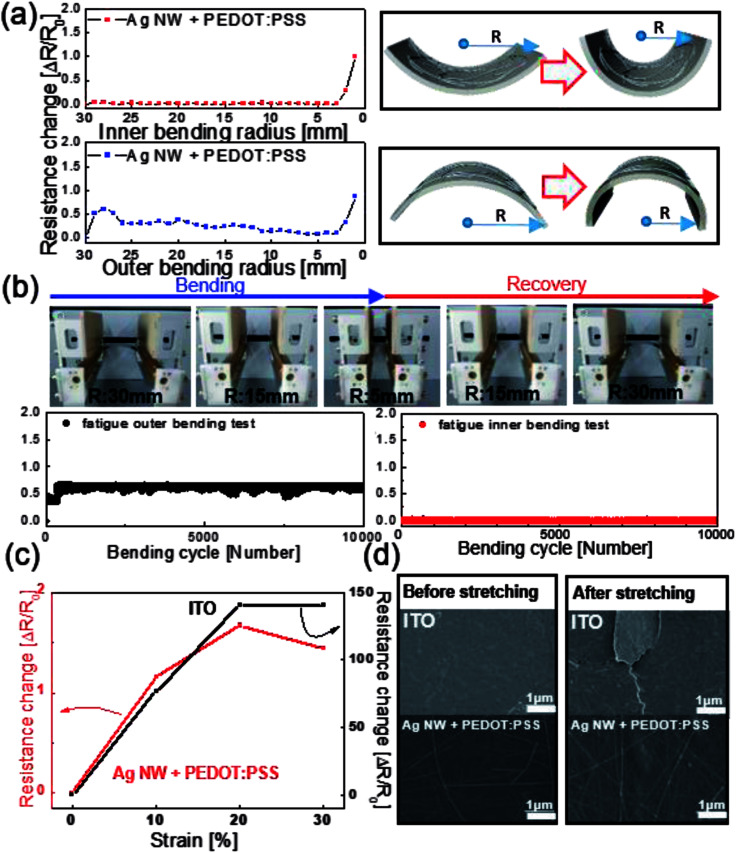
(a) Resistance change of the Ag NW/PEDOT:PSS hybrid TSEs with decreasing bending radius. Right panels show schematics of outer and inner bending. (b) Dynamic fatigue tests of the Ag NW/PEDOT:PSS hybrid TSE with increasing bending cycles at a fixed bending radius of 5 mm. The upper panels demonstrate the repeated bending cycles. (c) Stretching test results of the Ag NW/PEDOT:PSS hybrid TSE and ITO film on PU substrate. (d) Surface FESEM images of sputtered ITO and bar-coated Ag NW/PEDOT:PSS hybrid TSEs before and after 30% stretching.

We employed lab-designed bending tests to investigate the flexibility of bar-coated Ag NW/PEDOT:PSS hybrid TSEs. Fig. 5(a) shows the resistance change of the Ag NW/PEDOT:PSS hybrid electrode during outer and inner bending of the PU substrate with a decreasing bending radius. The right-side panels show a schematic of flexed Ag NW/PEDOT:PSS TSE samples with a decreasing bending radius. The change in the resistance (Δ*R*) of the measured samples can be expressed as (*R* − *R*_0_)/*R*_0_, where *R*_0_ is the resistance initially measured, and *R* is the resistance measured at a specific bending radius. Regardless of the bending mode, the Ag NW/PEDOT:PSS hybrid TSE showed a constant resistance change until a bending radius of 2 mm. Fig. 5(b) shows the results of repeated outer and inner bending testing at a constant bending radius of 5 mm and constant bending speed of 1 Hz. Upper panels show dynamic bending test steps using a lab-designed dynamic bending test machine. It was noteworthy that the Ag NW/PEDOT:PSS hybrid TSEs maintained their initial resistance for 10 000 cycles, indicating superior flexibility of Ag NW/PEDOT:PSS hybrid TSEs. Fig. 5(c) shows stretching test results of the Ag NW/PEDOT:PSS hybrid TSE and typical ITO coated on a PU substrate. ITO films showed an abrupt increase in resistance change above 10% stretching due to the brittleness of sputtered ITO films.^28–30^ However, the Ag NW/PEDOT:PSS hybrid TSE retained its initial sheet resistance (*R*_0_) even at a strain of 30%. Although the Ag NW/PEDOT:PSS electrode had a similar resistance in the as-coated state, the outstanding stretchability of the Ag NW/PEDOT:PSS hybrid films is critical for use as TSEs in the fabrication of stretchable PDLC-based smart windows. Fig. 5(d) shows surface FESEM images of the Ag NW/PEDOT:PSS and ITO film coated on a PU substrate before and after 30% stretching. After the stretching test, the ITO film showed the formation of cracks vertical to the stretching direction. The formation of cracks on ITO films led to the physical separation of the ITO film and an increase in resistance when the PU substrate was stretched. However, the Ag NW/PEDOT:PSS hybrid TSE showed a similar surface FESEM image even after 30% stretching, indicating superior stretchability against external stretching.

**Fig. 6:**
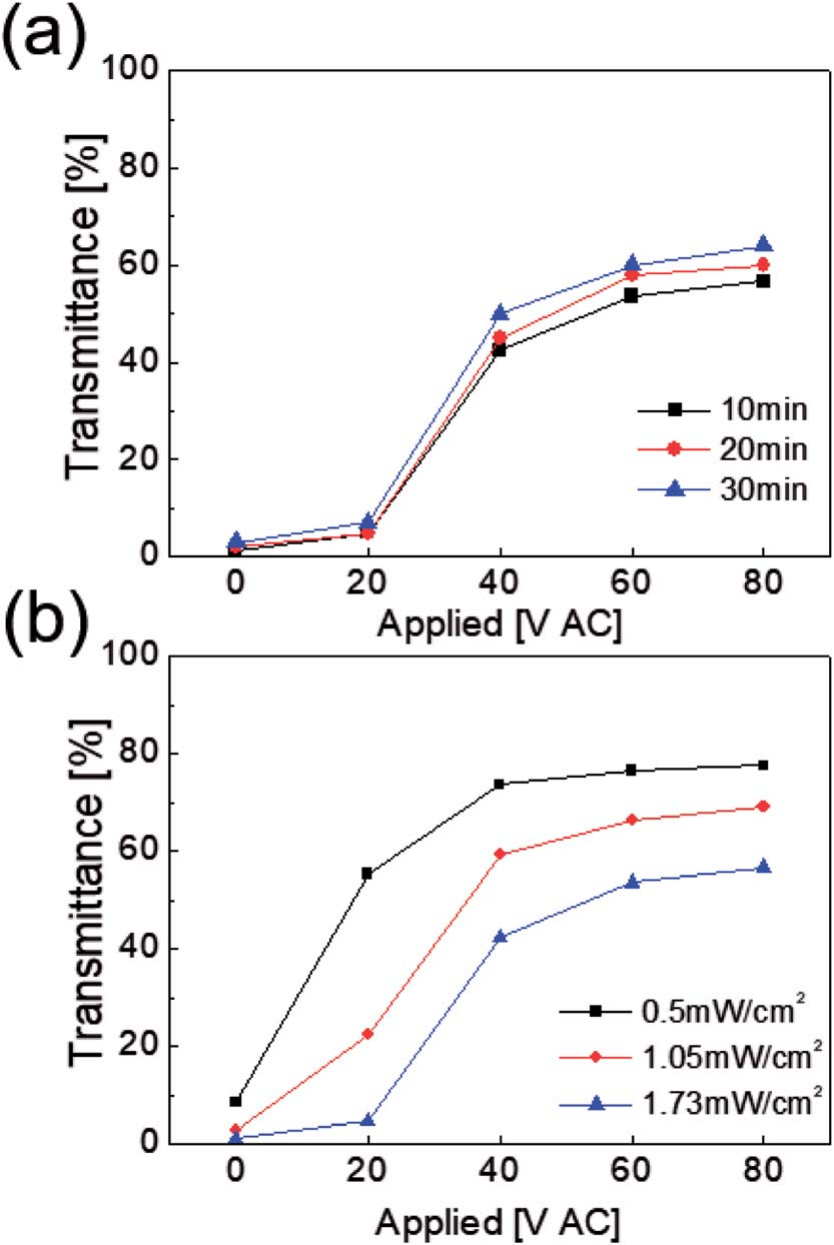
On-state transmittance of PDLC-based smart windows with Ag NW/PEDOT:PSS TSE (a) as a function of curing time and (b) UV light intensity.

To polymerize the polymer precursor of the PDLC solution, the bar-coated fluid homogeneous mixture in PDLC solution was irradiated with UV light (wavelength = 365 nm).^23^ Ahmad *et al.*, reported that PDLC-based smart windows have high on-state transmittance and contrast ratios (CR: the ratio of off transmittance and on transmittance of the PDLC smart window) under low UV intensity and long curing times.^31^ Therefore, we optimized curing time and UV intensity based on the optical transmittance of the PDLC-based smart window with the Ag NW/PEDOT:PSS hybrid TSEs. Fig. 6(a) shows the optical transmittance of the PDLC-based smart window with Ag NW/PEDOT:PSS hybrid TSEs as a function of curing time at a constant UV intensity of 0.5 mW cm^−2^. An increase in curing time led to an increase in on-state transmittance when the AC voltage (80 V) was applied. Fig. 6(b) also shows the on-state transmittance of the PDLC-based smart window with the Ag NW/PEDOT:PSS hybrid TSEs with increasing UV light irradiation intensity at a fixed curing time of 30 min. As shown, a decrease in UV light irradiation intensity led to an increase in on-state transmittance of the PDLC-based smart window due to less scattering for the sample cured at low intensity.^31^ Therefore, we fabricated a stretchable PDLC-based smart window at an optimal UV irradiation time and intensity of 30 minutes and 0.5 mW cm^−2^.

**Fig. 7:**
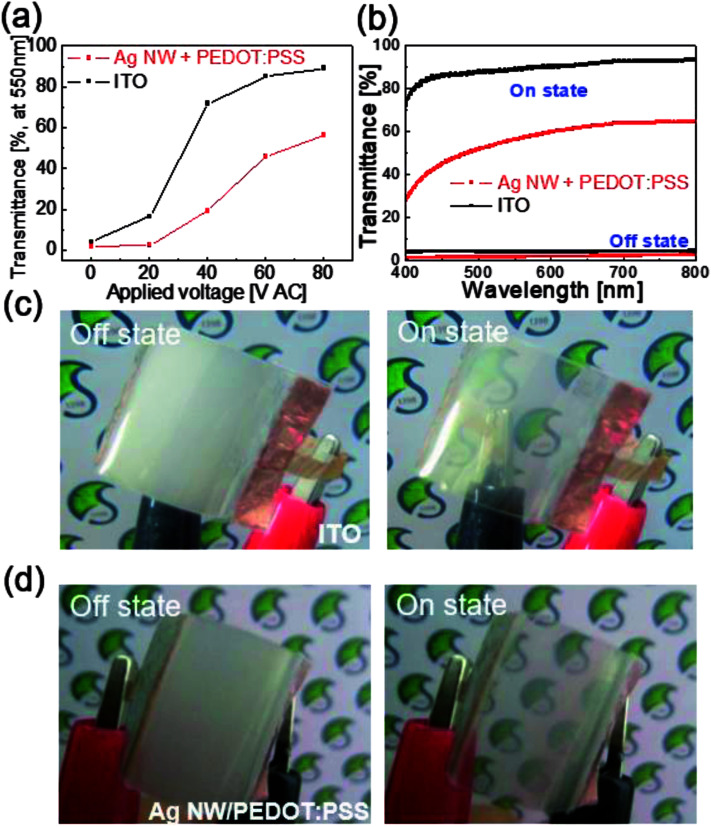
(a) On-state transmittance at a wavelength of 550 nm for the PDLC-based smart window with sputtered ITO electrodes and bar-coated Ag NW/PEDOT:PSS hybrid TSE on PU substrate. (b) Transmittance spectra of PDLC-based smart windows with Ag NW/PEDOT:PSS TSEs and ITO electrodes in the ‘on’ and ‘off’ states. Pictures of ‘off’ and ‘on’ state of PDLC-based smart windows with (c) ITO electrodes and (d) Ag NW/PEDOT:PSS hybrid TSEs with input AC voltage of 0 V (off state) and 80 V (on state) across the two Cu electrodes.

Fig. 7(a) compares the optical transmittance of the PDLC-based smart window with different ITO and Ag NW/PEDOT:PSS electrodes at a wavelength of 550 nm. Both PDLC-based smart window showed an increased on-state transmittance with increasing AC voltages. The on-state optical transmittance of the PDLC-based smart window with sputtered ITO electrodes was higher than that of the PDLC-based smart window with bar-coated Ag NW/PEDOT:PSS hybrid TSEs due to the higher optical transmittance of the ITO electrode. The on-state transmittance spectra and off-state transmittance spectra *versus* wavelength for both PDLC-based smart window are shown in Fig. 7(b). In the off-state, both PDLC-based smart window showed similar low transmittance due to light scattering in the PDLC layer. However, in the on-state, the PDLC-based smart window with sputtered ITO electrodes showed a higher optical transmittance in a visible wavelength region from 400 to 800 nm. The on-state transmittance of the PDLC-based smart window with ITO electrodes is about 90%, while the PDLC-based smart window with Ag NW/PEDOT:PSS hybrid TSEs is about 60%. Fig. 7(c) and (d) show the opaque and transparent images of flexed PDLC-based smart window with ITO electrodes and Ag NW/PEDOT:PSS hybrid TSEs in the off and on-states. Although the PDLC-based smart window with typical ITO electrodes showed a higher on-state transmittance than the PDLC-based smart window with Ag NW/PEDOT:PSS hybrid TSEs, it cannot be applied in stretchable PDLC-based smart window due to the brittleness of the ITO electrode.

**Fig. 8:**
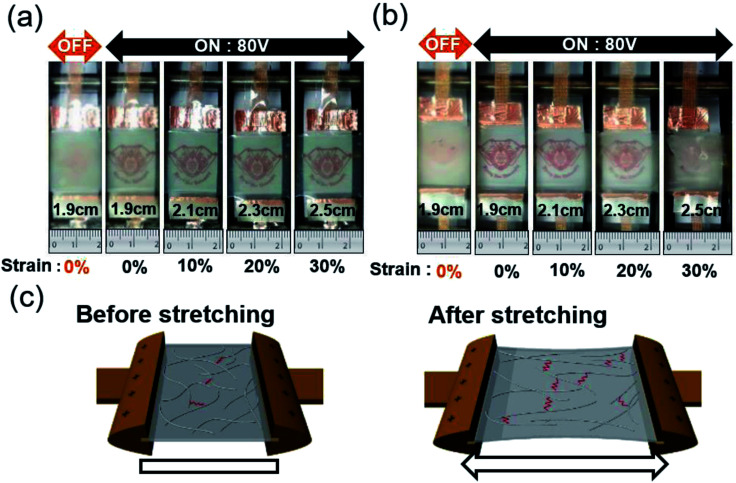
Pictures of (a) the Ag NW/PEDOT:PSS hybrid and (b) sputtered ITO based PDLC smart windows before and after sample stretching from 0% to 30%. At voltage-on state (80 V), both Ag NW/PEDOT:PSS based PDLC and ITO-based PDLC samples were stretched to 30% respectively. (c) Schematic of stretching mechanism for the Ag NW/PEDOT:PSS hybrid films.

We employed a lab-designed stretching tester shown in Fig. 8 to illustrate the better stretchability of Ag NW/PEDOT:PSS hybrid TSEs-based PDLC smart window than ITO electrodes-based PDLC smart window. At voltage-off state (0 V) and 0% strain, the PDLC is opaque with a typical color. The edge of the on-state PDLC-based smart window by applying a constant voltage of 80 V was clamped in the stretching tester and stretched up to 30%. Fig. 8(a) shows that Ag NW/PEDOT:PSS-based PDLC smart window was stretched from 0 to 30% without any abnormality due to the outstanding stretchability of the hybrid TSEs. However, the PDLC-based smart window with sputtered ITO electrodes became less transparent and eventually opaque after stretching to 30% as shown in Fig. 8(b). There was a disconnection in the ITO electrode when an AC voltage was applied to the PDLC layer due to the easy crack formation and fast propagation during stretching of the PDLC-based smart windows. For this reason, the on-state transmittance decreased when the sample stretched. Therefore, we confirmed that the Ag NW/PEDOT:PSS hybrid TSE-based PDLC smart window is effective in stretchable smart window. Fig. 8(c) demonstrates a possible mechanism to explain the outstanding stretchability of the Ag NW/PEDOT:PSS electrode coated on a PU substrate. In the as-coated sample, the Ag NW network acts as a main current path and therefore showed a low sheet resistance. When the sample was severely stretched, some Ag NWs were connected, but some Ag NWs were disconnected. However, the conductive PEDOT:PSS matrix still maintained contact between the disconnected Ag NWs. Therefore, the hybrid electrode could maintain its low resistance as illustrated in Fig. 8(c). This effect is referred to as the bridge effect of the conductive PEDOT:PSS matrix.^22,32^ Like conductive graphene in graphene and Ag NW hybrid electrodes, the conductive PEDOT:PSS electrode connected the short Ag NWs to allow for current flow.^33^

**Fig. 9:**
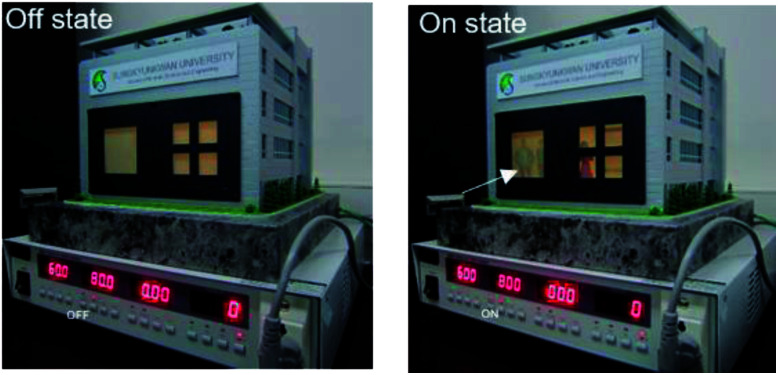
Promising application of Ag NW/PEDOT:PSS hybrid TSEs-based PDLC smart window for smart building.

Fig. 9 demonstrates promising application of the Ag NW/PEDOT:PSS hybrid TSEs based-PDLC smart window. It can be employed in smart windows for next generation smart buildings or automobiles. The operation of the smart window was conducted at a frequency of 60 Hz and a voltage of 80 V. When there is no voltage between TSEs of smart windows, building can be kept in a private. On the other hand, it appears clearly transparent at an applied voltage (80 V), and we can see people inside the building as indicated by arrows. Based the low sheet resistance, high transparency, simple coating process and superior flexibility and stretchability of the Ag NW/PEDOT:PSS film, we expect high performance stretchable PDLC-based smart windows with fast on-off transparency change, good flexibility that are cost-effective over large areas. Although there is no commercial PDLC-based stretchable smart window at this moment, the PDLC-based stretchable smart windows could be applied in some light shadowing stretchable window in automobiles or light controllable window for wearable devices in the near future.

## 4. Conclusions

We developed a solution-processed Ag NW/PEDOT:PSS hybrid TSEs for stretchable PDLC-based smart window by using Ag NW/PEDOT:PSS mixed ink. We used simple bar coating of Ag NWs and PEDOT:PSS mixed ink on a PU substrate to fabricate highly stretchable and transparent hybrid TSEs. These TSEs showed a sheet resistance of 40 ohm per square, an optical transmittance of 82%, and stretchability of 30%, making them candidates to replace conventional brittle ITO electrodes. We conducted bending and stretching tests to prove the outstanding mechanical properties of Ag NW and PEDOT:PSS hybrid TSE. Ag NW/PEDOT:PSS hybrid films were employed as the TSE in PDLC-based stretchable smart windows, which cannot be realized using brittle ITO-based smart windows. The stretchable PDLC-based smart window exhibited an on-state transmittance of 56% at an applied voltage of 80 V and an off-state transmittance of 2% at 0 voltage. Unlike the ITO-based PDLC smart window, which is easily broken by stretching, the Ag NW/PEDOT:PSS hybrid electrode-based PDLC smart window was stretched up to 30%. Successful operation of the stretchable PDLC-based smart window indicates that Ag NW/PEDOT:PSS hybrid films are promising TSEs for cost-effective, large area, and stretchable smart windows for use in next generation smart buildings and automobiles.

## Conflicts of interest

The authors declare no competing financial interests.

## Supplementary Material

## References

[cit1] Su C.-W., Chen M.-Y. (2014). J. Disp. Technol..

[cit2] Resetic A., Milavec J., Zupancic B., Domenici V., Zalar B. (2016). Nat. Commun..

[cit3] Su C.-W., Liao C.-C., Chen M.-Y. (2016). J. Disp. Technol..

[cit4] Kim M., Park K. J., Seok S., Ok J. M., Jung H.-T., Choe J., Kim D. H. (2015). ACS Appl. Mater. Interfaces.

[cit5] Vaz N. A. and Montgomery G. P., Liquid Crystal Materials, Devices, and Applications, 1992, p. 1665

[cit6] Nishimura E., Sasabayashi T., Ito N., Sato Y., Utsumi K., Yano K., Kaijo A., Inoue K., Shigesato Y. (2007). J. Appl. Phys..

[cit7] Boehme M., Charton C. (2005). Surf. Coat. Technol..

[cit8] Sierros K.-A., Morris N.-J., Ramji K., Cairns D.-R. (2009). Thin Solid Films.

[cit9] Kim Y., Kim K., Kim K. B., Park J.-Y., Lee N., Seo Y. (2016). Curr. Appl. Phys..

[cit10] Chung S.-H., Noh H. Y. (2015). Opt. Express.

[cit11] Liu Y., Shen S., Hu J., Chen L. (2016). Opt. Express.

[cit12] Khaligh H.-H., Liew K., Han Y., Abukhdeir N.-M., Goldthorpe I.-A. (2015). Sol. Energy Mater. Sol. Cells.

[cit13] Chen T. L., Ghosh D. S., Mkhitaryan V., Pruneri V. (2013). ACS Appl. Mater. Interfaces.

[cit14] Kim E. M., Choi I.-S., Oh J.-P., Kim Y.-B., Lee J.-H. (2014). Jpn. J. Appl. Phys..

[cit15] Seo K.-W., Lee J.-H., Cho N.-K., Kang S.-J., Na S.-I., Kim H.-K. (2014). J. Vac. Sci. Technol., A.

[cit16] Seo K.-W., Lee J.-H., Kim H.-J., Na S.-I., Kim H.-K. (2014). Appl. Phys. Lett..

[cit17] Kim D.-H., Ko E.-H., Kim K.-H., Kim T.-W., Kim H.-K. (2016). ECS J. Solid State Sci. Technol..

[cit18] Park J.-Y., Hong S., Jang J., Kim H.-K. (2017). ECS J. Solid State Sci. Technol..

[cit19] Lee S.-M., Kim S.-H., Lee J.-H., Kim H.-K. (2018). RSC Adv..

[cit20] Seo K.-W., Kim H.-K. (2015). Thin Solid Films.

[cit21] Jin Y., Deng D., Cheng Y., Kong L., Xiao F. (2014). Nanoscale.

[cit22] Lim J.-E., Lee S.-M., Kim S.-S., Koo T.-W., Kim H.-K. (2017). Sci. Rep..

[cit23] Jin J.-M., Parbhakar K., Dao L. H. (1995). Liq. Cryst..

[cit24] Wang P.-C., Macdiarmid A. G. (2007). Displays.

[cit25] Cupelli D., Nicoletta F. P., Manfredi S., Vivacqua M., Formoso P., Filpo G. D., Chidichimo G. (2009). Sol. Energy Mater. Sol. Cells.

[cit26] Yang K.-J., Kang J.-K., Choi B.-D. (2014). Jpn. J. Appl. Phys..

[cit27] Long L., Ye H. (2014). Sci. Rep..

[cit28] Lee S.-M., Koo H.-W., Kim T.-W., Kim H.-K. (2018). Surf. Coat. Technol..

[cit29] Choi K.-H., Kim J., Noh Y.-J., Na S.-I., Kim H.-K. (2013). Sol. Energy Mater. Sol. Cells.

[cit30] Kang S.-B., Kim H.-K. (2014). Thin Solid Films.

[cit31] Ahmad F., Jamil M., Lee J.-W., Kim S.-R., Jeon Y.-J. (2016). Electron. Mater. Lett..

[cit32] Wei Y., Chen S., Dong X., Lin Y., Liu L. (2017). Carbon.

[cit33] Lee J.-H., Shin H.-S., Noh Y.-J., Na S.-I., Kim H.-K. (2013). Sol. Energy Mater. Sol. Cells.

